# Supplementary material for: Monosymmetric Fe-N4 sites enabling durable proton exchange membrane fuel cell cathode by chemical vapor modification
Source: Nat Commun. 2024 May 17;15:4219. doi: 10.1038/s41467-024-47817-0 (PMC11101623; doi:10.1038/s41467-024-47817-0)
Supplement: Supplementary file 1 — Supplementary Information [file 41467_2024_47817_MOESM1_ESM.pdf]

Monosymmetric $Fe-N_4$ sites enabling durable proton exchange membrane fuel cell cathode by chemical vapor modification

Jingsen Bai[†, 1, 2], Tuo Zhao[†, 3], Mingjun Xu[†, 1,2], Bingbao Mei[†, 4], Liting Yang[1, 2], Zhaoping Shi[1, 2], Siyuan Zhu[1, 2], Ying Wang*[, 5], Zheng Jiang[6], Zhao Jin*[,1, 2], Junjie Ge*[,1, 2], Meiling Xiao[1, 2], Changpeng Liu[1, 2], Wei Xing*[,1, 2]

[1]State Key Laboratory of Electroanalytic Chemistry, Jilin Province Key Laboratory of Low Carbon Chemistry Power, Changchun Institute of Applied Chemistry, Chinese Academy of Sciences, Changchun 130022, China.
[2]School of Applied Chemistry and Engineering, University of Science and Technology of China, Hefei 230026, China.
[3]Commercial Vehicle Development Institute, FAW Jiefang Automotive CO.LTD., Changchun 130011, China.
[4]Shanghai Synchrotron Radiation Facility, Shanghai Advanced Research Institute, Chinese Academy of Sciences, Shanghai 201800, PR China.
[5]State Key Laboratory of Rare Earth Resource Utilization, Changchun Institute of Applied Chemistry, Chinese Academy of Sciences, Changchun 130022, China.
[6]National Synchrotron Radiation Laboratory (NSRL), University of Science and Technology of China, Hefei 230026, China.
†These authors contributed equally.
*Corresponding author email: ywang_2012@ciac.ac.cn; zjin@ciac.ac.cn; gejunjie@ustc.edu.cn; xingwei@ciac.ac.cn

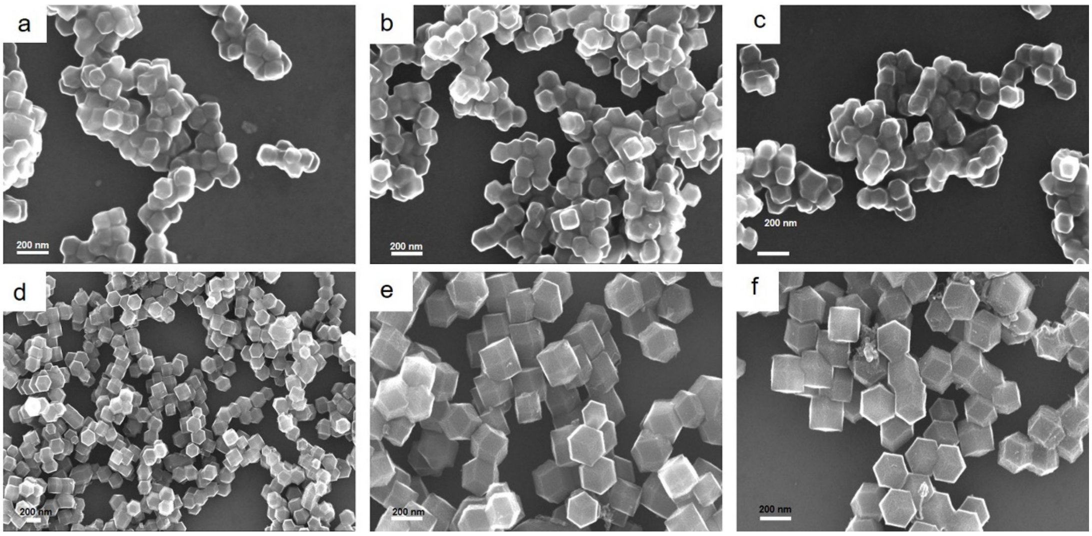

Supplementary Figure 1. | Morphology characterization on electrocatalysts. SEM images of (a-c) Fe-N-C and (d-e) Fe-N-C_{CVM}.

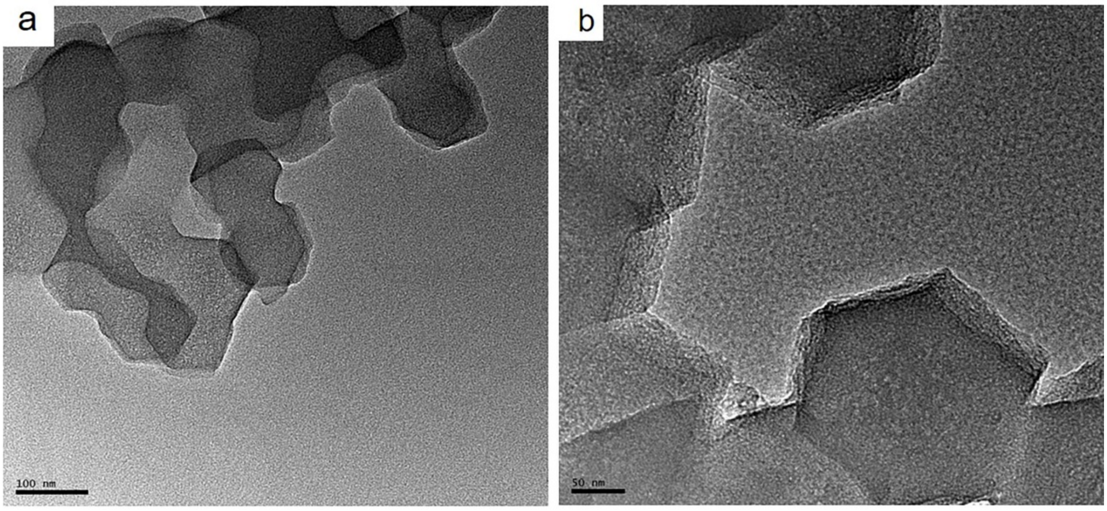

Supplementary Figure 2. | Morphology characterization on electrocatalysts. TEM images of (a) Fe-N-C and (b) Fe-N-C_{CVM}.

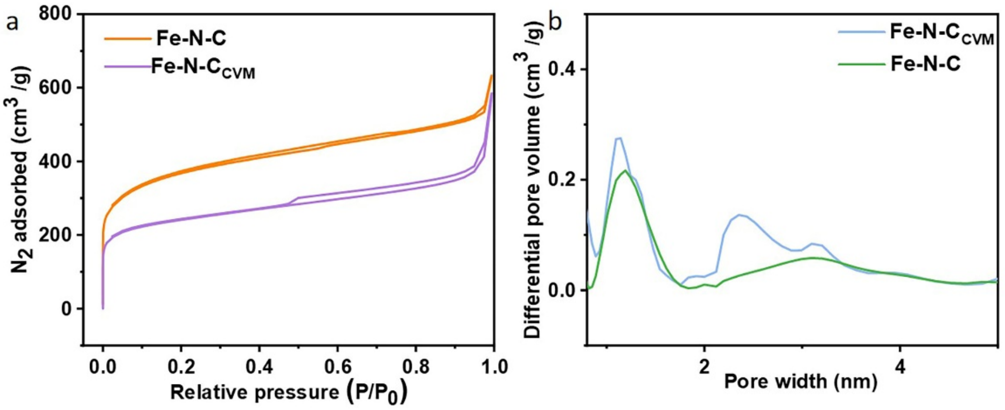

Supplementary Figure 3. | Nitrogen adsorption/desorption isotherm measurements. (a) N_2 adsorption/desorption and (b) pore distribution plots of Fe-N-C and Fe-N-C$_{CVM}$.

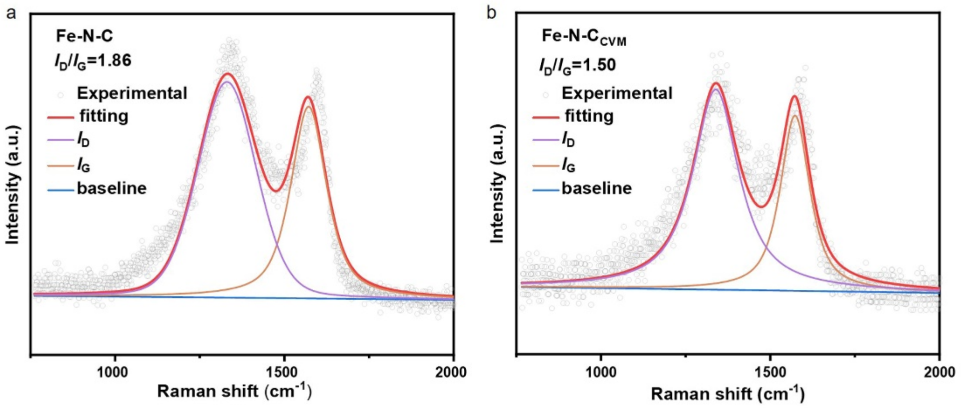

Supplementary Figure 4. | Evaluation of the graphitization degree of the two electrocatalysts. Raman spectra of the Fe-N-C and Fe-N-C_{CVM} electrocatalysts.

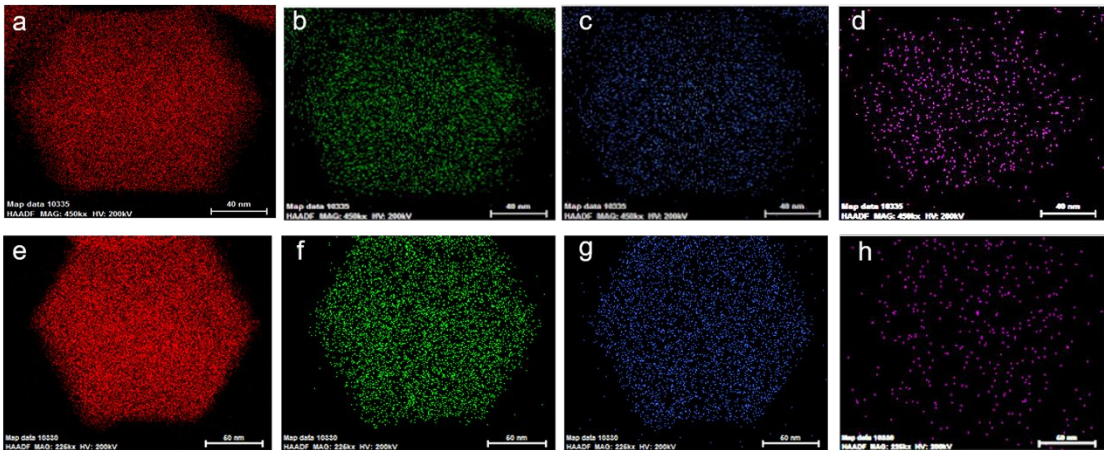

Supplementary Figure 5. | Elemental distribution tests. Energy dispersive X-ray spectroscopy (EDS) maps of C (a,e), N (b,f), O (c,g), Fe (d,h) of electrocatalysts after and before chemical vapor etching, respectively.

Supplementary Table 1 Zn and Fe contents in electrocatalysts determined by ICP-MS

Samples	Elements	Content (wt. %)
Fe-N-C	Fe	0.63
	Zn	0.1
Fe-N-C_{CVM}	Fe	1.13
	Zn	0.08

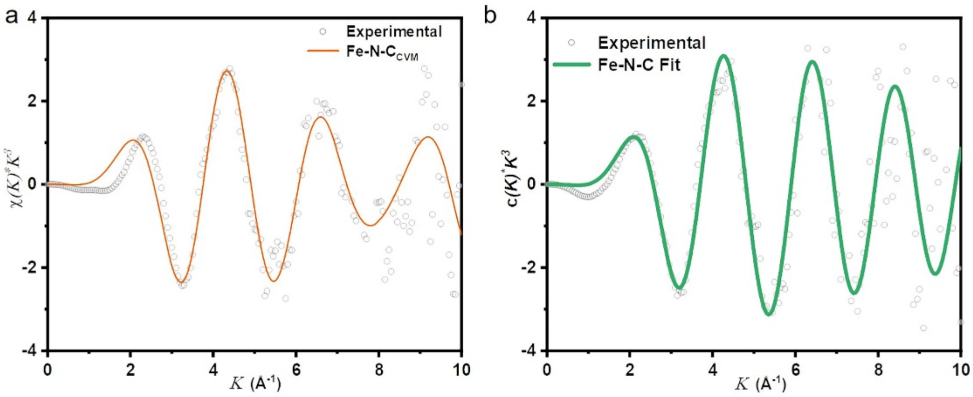

Supplementary Figure 6. | K-space EXAFS spectra. (a) EXAFS fitting of Fe-N-C$_{CVM}$ in *k* space. (b) EXAFS fitting of Fe-N-C in *k* space.

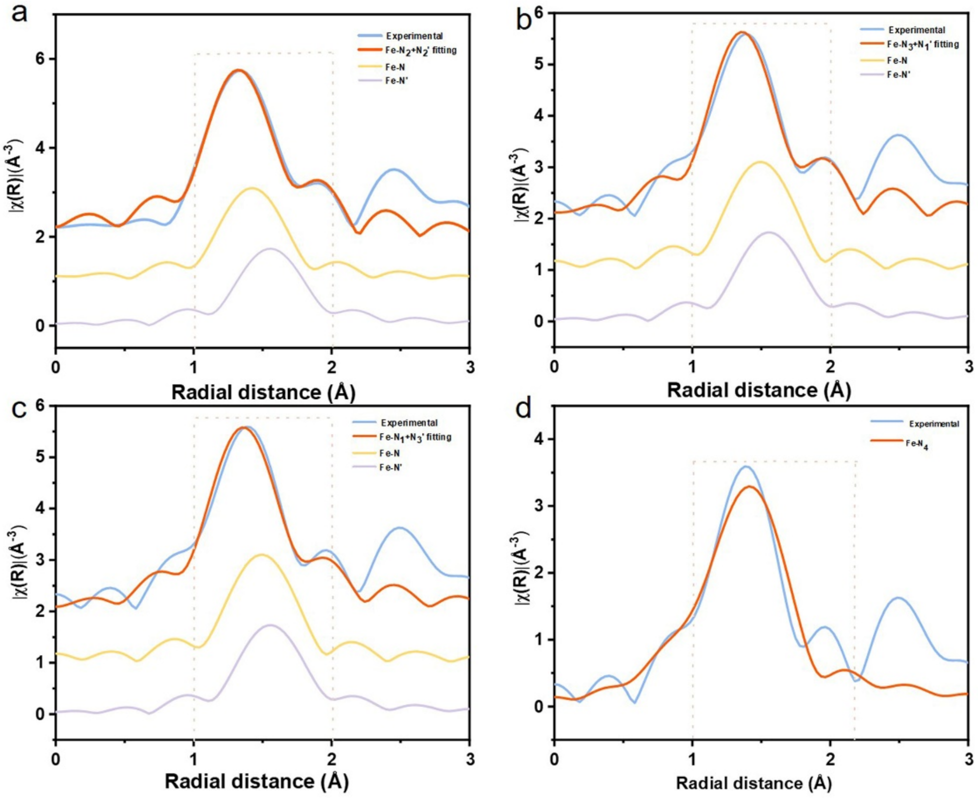

Supplementary Figure 7. | Fine structure analysis. Ex situ Fe K-edge Fourier transform EXAFS spectrum and its fitting with (a) the coordination number evenly distributed in the two paths, (b) first path filled 3 N atoms, the other path filled 1 N atom, (c) first path filled 1, the other path filled 3 N atoms and (d) four coordination atoms completely filled in a path of Fe-N-C_{CVM}.

Supplementary Table 2 Results of the fitting of the FT-EXAFS spectra collected at the Fe-Kedge for Fe-N-C_{CVM} (CN: coordination number; R: distance; σ^2: mean-square disorder; ΔE_0: energy shift).

path	CN	R (Å)	ΔE_0 (eV)	$\sigma^2 \times 10^{-3}$ (Å^2)	R factor (%)
Fe-N_2	2	1.87(5)	0.02	2.3	0.021
Fe-N_2’	2	2.06	1.51	4.4	0.029

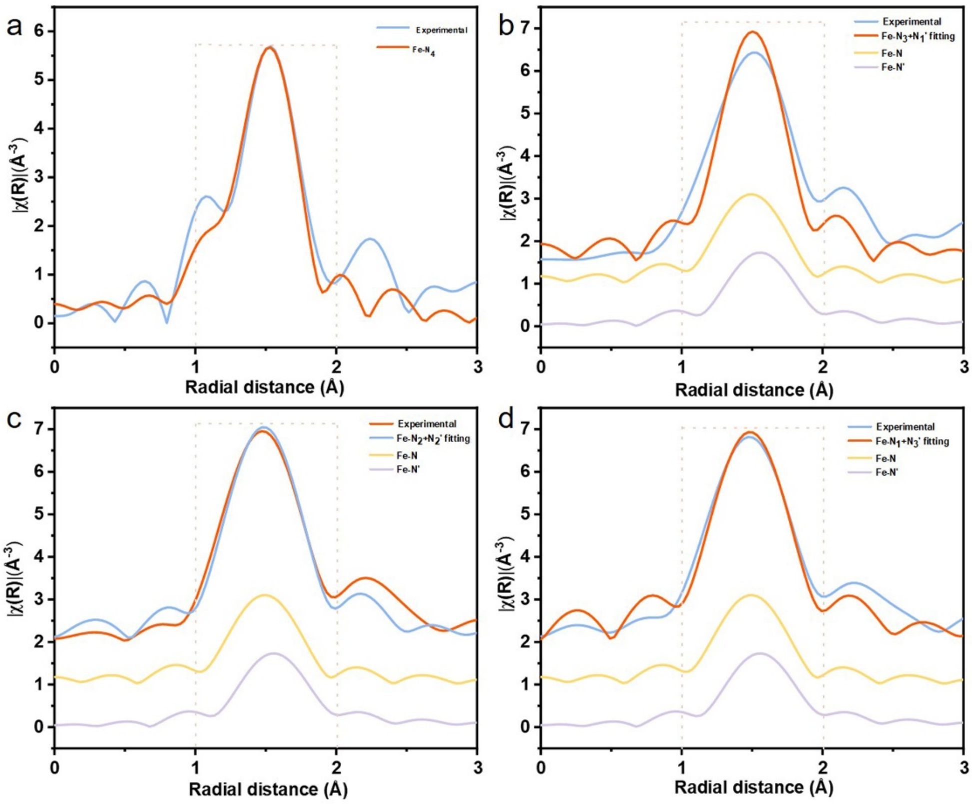

Supplementary Figure 8. | Fine structure analysis. Ex situ Fe K-edge Fourier transform EXAFS spectrum and its fitting with (a) four coordination atoms completely filled in a path, (b) first path filled 3 N atoms, the other path filled 1 N atom, (c) the coordination number unevenly distributed in the two paths and (d) first path filled 1, the other path filled 3 N atoms of Fe-N-C.

Supplementary Table 3 Results of the fitting of the FT-EXAFS spectra collected at the Fe-Kedge for Fe-N-C (CN: coordination number; R: distance; σ^2: mean-square disorder; ΔE_0: energy shift).

path	CN	R (Å)	ΔE_0 (eV)	$\sigma^2 \times 10^{-3}$ (Å^2)	R factor (%)
$Fe\text{-}N_4$	4	1.96 (9)	0.173	3.4	0.038

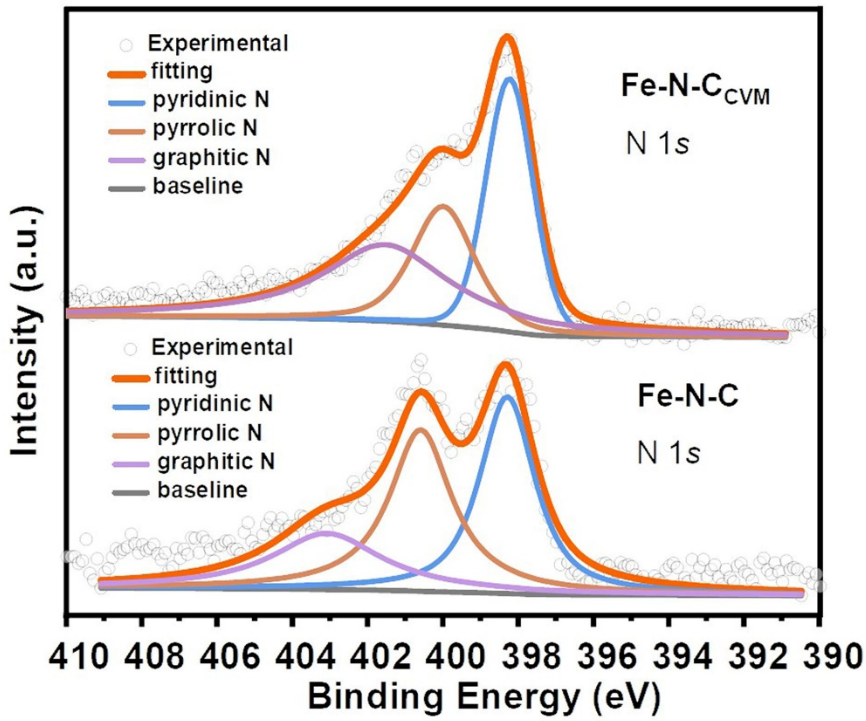

Supplementary Figure 9. | N 1*s* XPS spectra. High-resolution N 1s XPS data of the Fe-N-C and Fe-N-C$_{CVM}$ electrocatalysts.

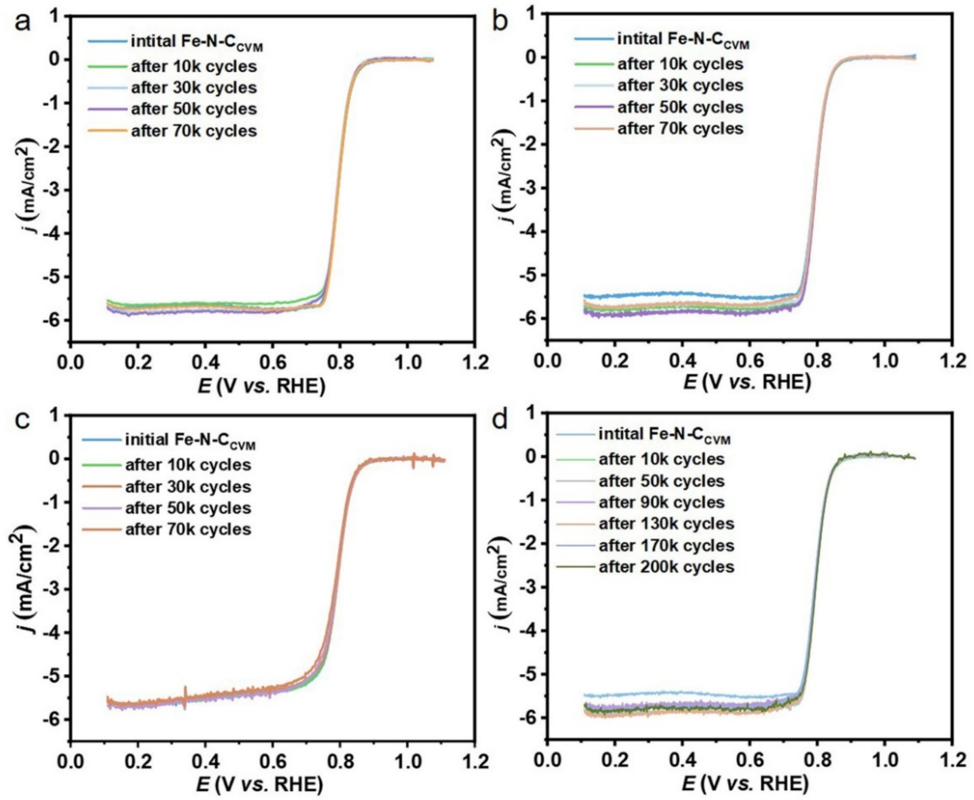

Supplementary Figure 10. | Durability tests. (a-d) ORR polarization curves of different batches of Fe-N-C_{CVM} before and after different potential cycles between 0.6-1.0V.

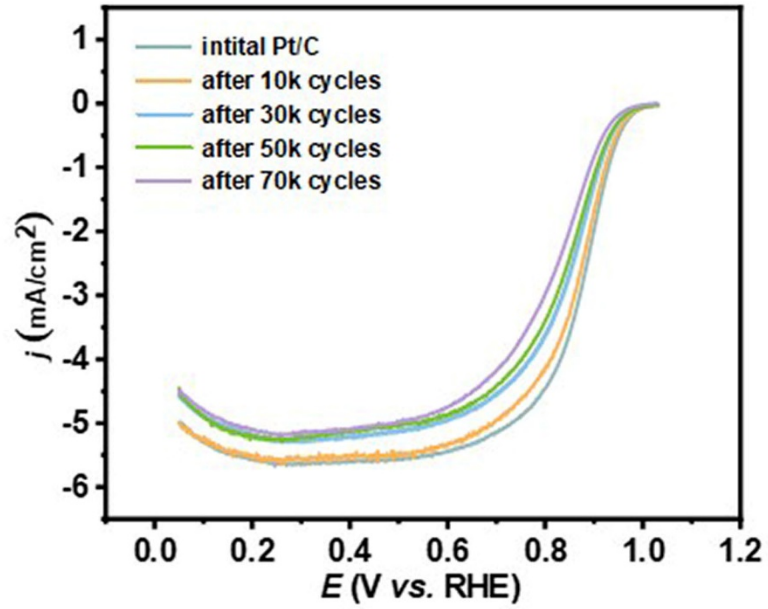

Supplementary Figure 11. | Durability tests. ORR polarization curves of Pt/C before and after different potential cycles between 0.6-1.0V.

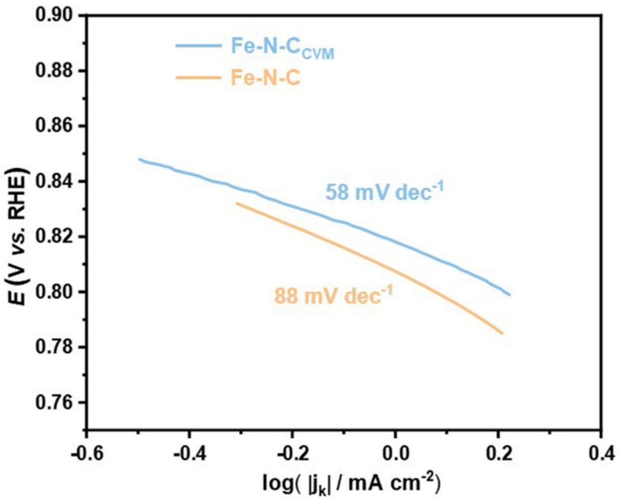

Supplementary Figure 12. | Tafel plots. Tafel plots for Fe-N-C$_{CVM}$ and Fe-N-C.

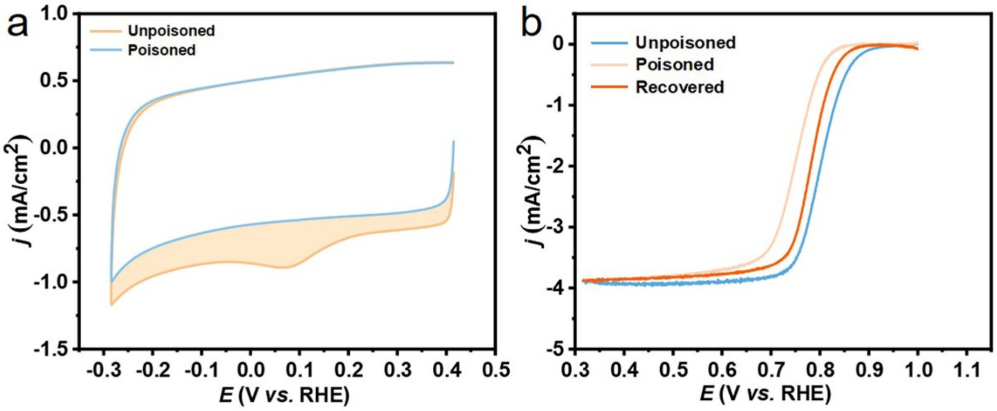

Supplementary Figure 13. | Turnover frequency tests. (a) Nitrite stripping voltammetry of Fe-N-C in N_2-saturated 0.5 M acetate electrolyte buffer (pH 5.2) with an electrocatalyst loading of 270 μg cm^{-2} and a scan rate of 10 mV s^{-1}. (b) ORR polarization curves of initial Fe-N-C in O_2-saturated 0.5 M acetate electrolyte buffer (pH 5.2) with an electrocatalyst loading of 270 μg cm^{-2} and a scan rate of 10 mV s^{-1}.

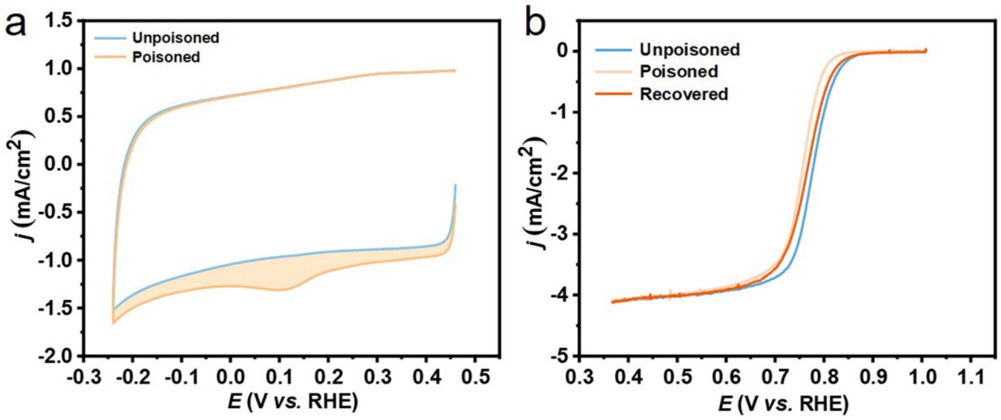

Supplementary Figure 14. | Turnover frequency tests. (a) Nitrite stripping voltammetry of Fe-N-C after 70,000 cycles ASTs in N_2-saturated 0.5 M acetate electrolyte buffer (pH 5.2) with an electrocatalyst loading of 270 µg cm^{-2} and a scan rate of 10 mV s^{-1}. (b) ORR polarization curves of initial Fe-N-C after ASTs in O_2-saturated 0.5 M acetate electrolyte buffer (pH 5.2) with an electrocatalyst loading of 270 µg cm^{-2} and a scan rate of 10 mV s^{-1}.

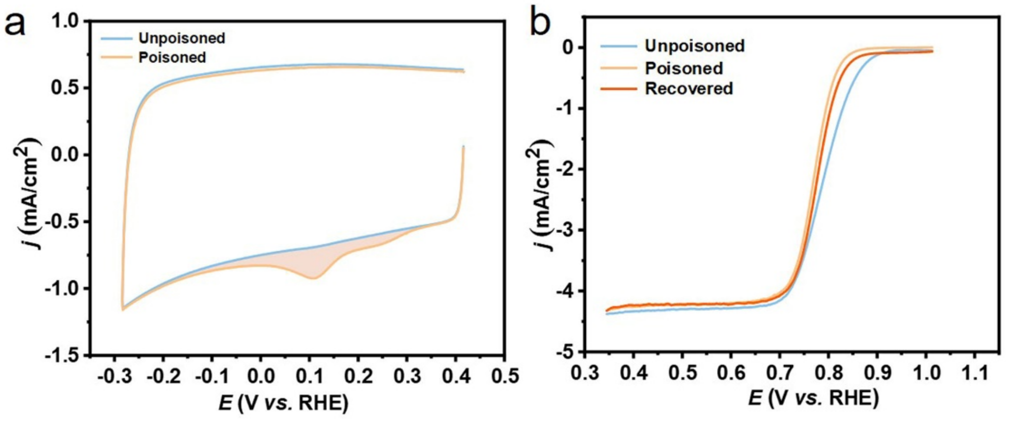

Supplementary Figure 15. | Turnover frequency tests. (a) Nitrite stripping voltammetry of initial Fe-N-C_{CVM} in N_2-saturated 0.5 M acetate electrolyte buffer (pH 5.2) with an electrocatalyst loading of 270 μg cm^{-2} and a scan rate of 10 mV s^{-1}. (b) ORR polarization curves of initial Fe-N-C_{CVM} in O_2-saturated 0.5 M acetate electrolyte buffer (pH 5.2) with an electrocatalyst loading of 270 μg cm^{-2} and a scan rate of 10 mV s^{-1}.

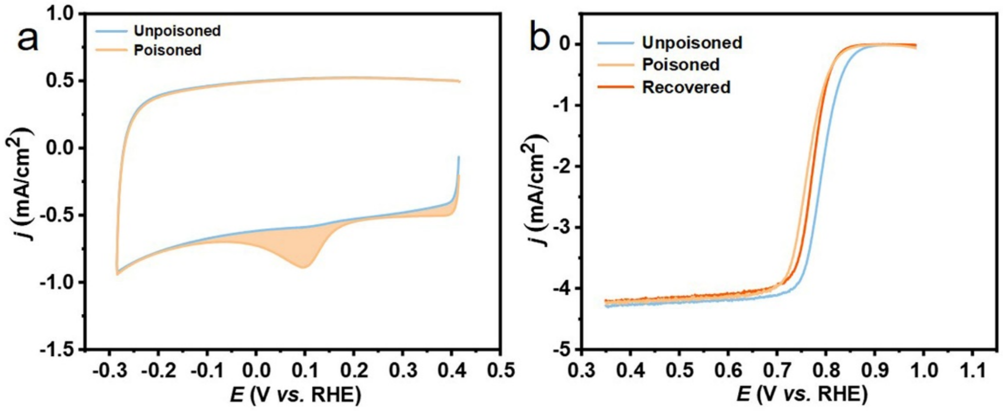

Supplementary Figure 16. | Turnover frequency tests. (a) Nitrite stripping voltammetry of Fe-N-C_{CVM} after 70,000 cycles ASTs in N_2-saturated 0.5 M acetate electrolyte buffer (pH 5.2) with an electrocatalyst loading of 270 µg cm^{-2} and a scan rate of 10 mV s^{-1}. (b) ORR polarization curves of initial Fe-N-C_{CVM} after ASTs in O_2-saturated 0.5 M acetate electrolyte buffer (pH 5.2) with an electrocatalyst loading of 270 µg cm^{-2} and a scan rate of 10 mV s^{-1}.

Supplementary Table 4 The $E_{1/2}$ loss of various electrocatalysts during ASTs.

Electrocatalyst	$E_{1/2}$ loss after 10k cycles, 30k cycles, and 50k cycles (mV)	Reference
20Fe-NC-second	/ 29 /	Nature Catalysis 2018, 1 (12), 935-945[1]
Fe/N/C-950	/ 31 /	Angew. Chem. Int. Ed. Engl. 2019, 58 (36), 12469-12475[2]
ZIF-NC-0.5Fe-700<br>ZIF-NC-0.5Fe-400	/ 31 /<br>/ 40 /	Angew. Chem. Int. Ed. Engl. 2019, 58 (52), 18971-18980[3]
Fe–N–C	40 / /	Nature Energy 2022, 7 (3), 281-289[4]
Fe-N-C	21 45 /	Chem Catalysis 2023, 3 (3)[5]
Fe-N-C-Phen-PANI	18 / /	Adv. Mater. 2017, 29 (7)[6]

Supplementary Table 5 Fe, N and O contents in electrocatalysts determined by EDS.

Samples	Fe (wt %)	N (wt %)	O (wt %)
Fe-N-C	0.16	3.58	2.09
Fe-N-C after AST	0.11	3.79	6.39
Fe-N-C_{CVM}	0.35	4.62	2.08
Fe-N-C_{CVM} after AST	0.33	4.52	1.74

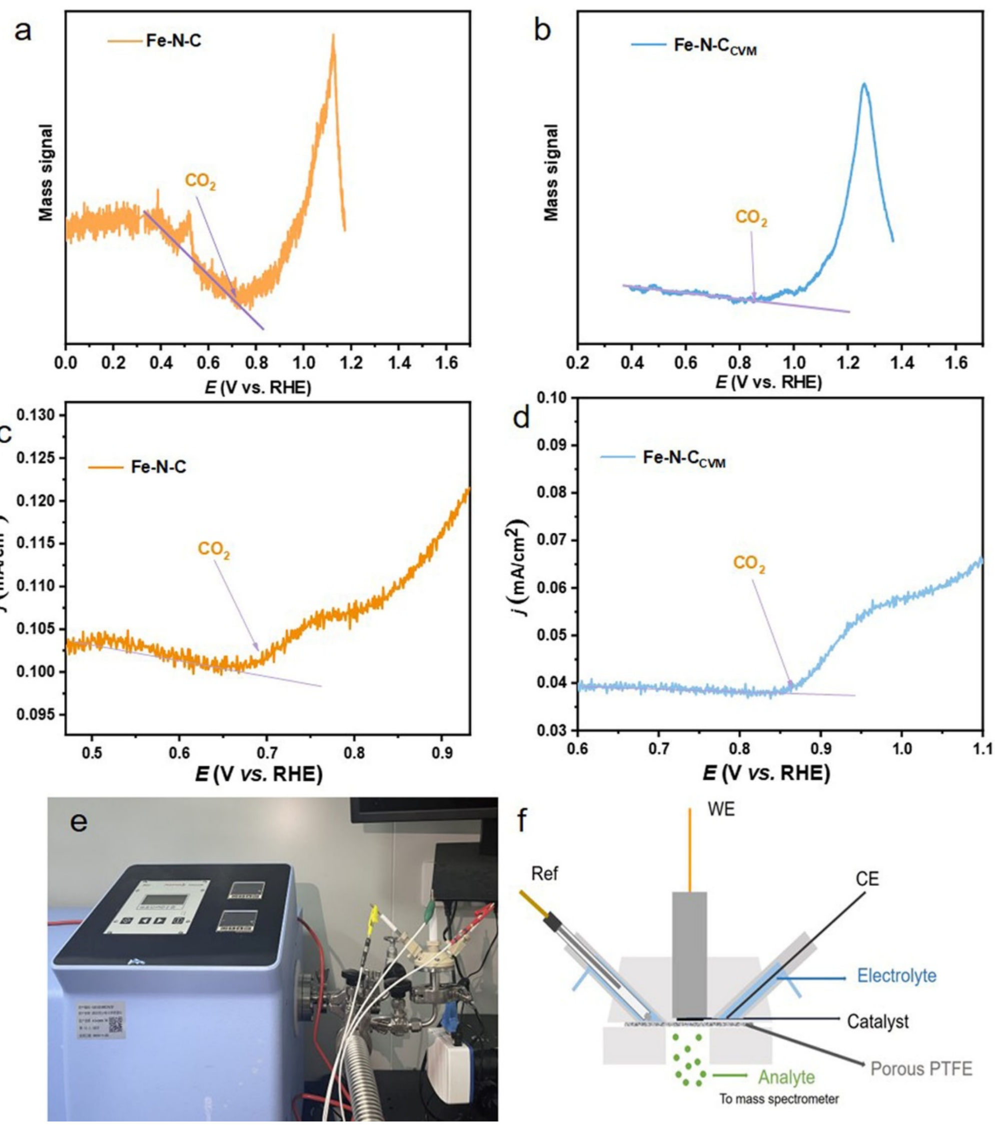

Supplementary Figure 17. | *In situ* DEMS results of Fe-N-C and Fe-N-C$_{CVM}$. DEMS signals of CO_2 from the reaction products for (a) Fe-N-C and (b) Fe-N-C$_{CVM}$ in 0.1M $HClO_4$. (c) The voltaic curve of Fe-N-C. (d) The voltaic curve of Fe-N-C$_{CVM}$. (e) Optical photo of the DEMS system. (f) schematic diagram of the electrochemical cell configuration used in DEMS measurements.

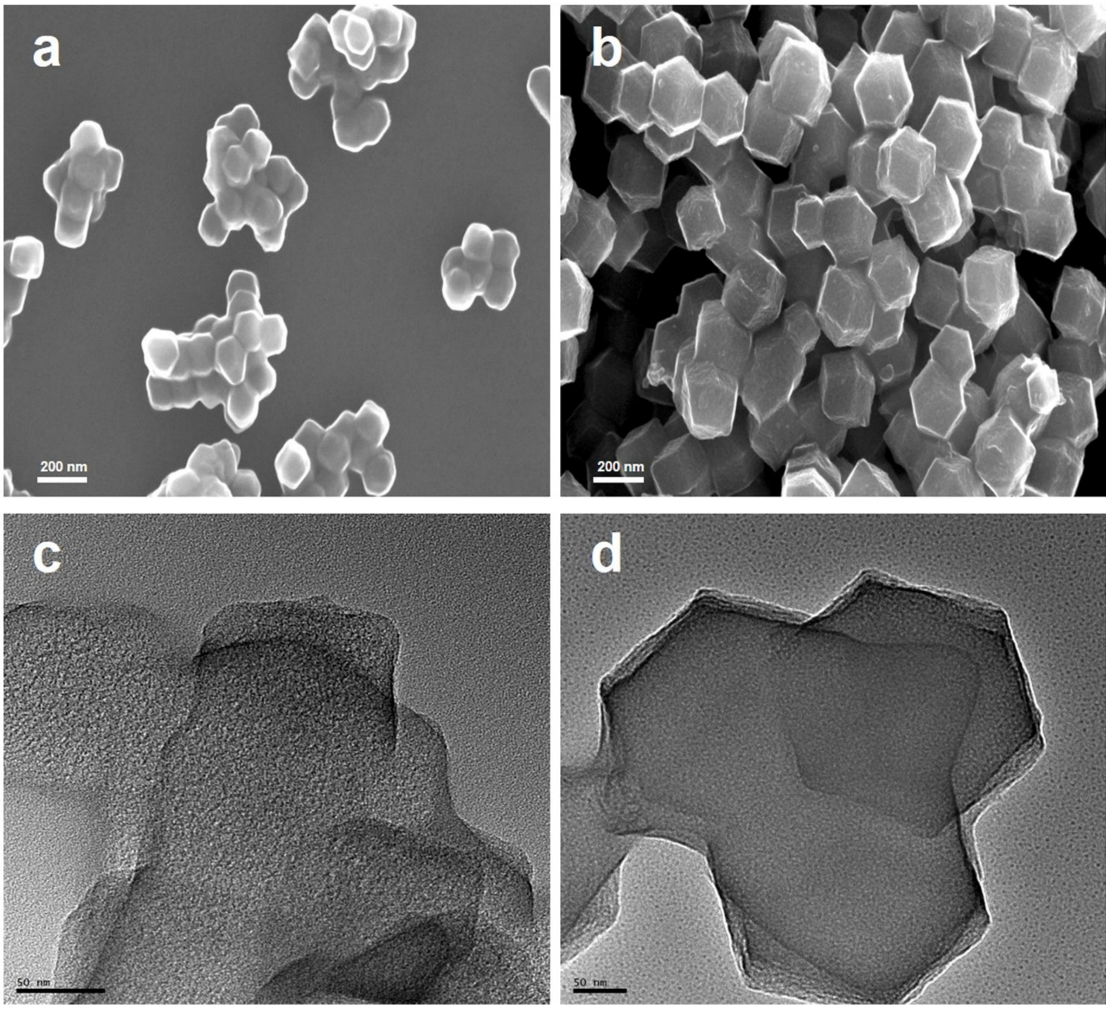

Supplementary Figure 18. | Morphology characterization on Fe-N-C and Fe-N-C$_{CVM}$. SEM images of the Fe-N-C electrocatalyst (a) and the Fe-N-C$_{CVM}$ electrocatalyst(b). TEM images of the Fe-N-C electrocatalyst (c) and the Fe-N-C$_{CVM}$ electrocatalyst(d).

Supplementary Table 6 Comparisons of the ORR performance in MEA-level under 1 bar H_2-air in recently published papers.

Electrocatalyst	Power density (mW cm^{-2})	Reference
TPI@Z8(SiO_2)-650-C	420	Nat catalysis 2019 2 (3), 259-268[7]
$FeCl_2$\/NC-1000	280	J. Am. Chem. Soc. 2020, 142 (3), 1417-1423[8]
Co(mIm)–NC (1.0)	320	Nature Catalysis 2020, 3 (12), 1044-1054[9]
FeNC-CVD-750	370	Nat Mater 2021, 20 (10), 1385-1391[10]
20Co-NC-1100	280	Adv. Mater. 2018, 30 (11)[11]
Fe-AC-AC-CVD	560	Nature Energy 2022, 7 (7), 652-663[12]
ZIF-NC-0.5Fe-700	290	Angew. Chem. Int. Ed. Engl. 2019, 58 (52), 18971-18980[3]
20-Fe NC-second	240	Nature Catalysis 2018, 1 (12), 935-945[1]
Fe-N-C-Phen-PANI	380	Adv. Mater. 2017, 29 (7)[6]
Fe SAs/N−C	350	ACS Catalysis 2019, 9 (3), 2158-2163.[13]

Supplementary Table 7 Comparisons of the Long-term fuel cell tests in MEA-level under 1 bar H_2-air in recently published papers.

Electrocatalyst	Operational duration	Test conditions	Reference
0.17/Fe-N-C-kat	170 h	H-Air 0.7V	Angew. Chem. Int. Ed. 2020, 59, 21698-21705[14]
20Co-NC-1100	100 h	H-Air 0.7V	Adv. Mater. 2018, 30, 1706758[11]
Fe-N-C	25 h	H-Air 0.7V	Adv. Mater. 2019, 31, 1807615[15]
Co(mIm)-NC	100 h	H-Air 0.7V	Nature Catalysis volume 3, 1044-1054 (2020)[9]
Fe_{SA}/FeAC-2DNPC	150 h	H-Air 0.5V	Nat Commun 13, 2963 (2022)[16]
Fe_g-NC/Phen	25 h	H-Air 06V	Energy Environ. Sci., 2022,15, 3033-3040[17]
Fe-AC-CVD	320 h	H-Air 0.67V	Nature Energy volume 7, 652-663 (2022)[12]
Fe-NC/Sca	22 h	H-Air	Angew. Chem.Int. Ed.2023,62, e2023061[18]
ma-Co-NC	100 h	H-Air 0.7V	Applied Catalysis B: Environmental 308 (2022) 121220[19]

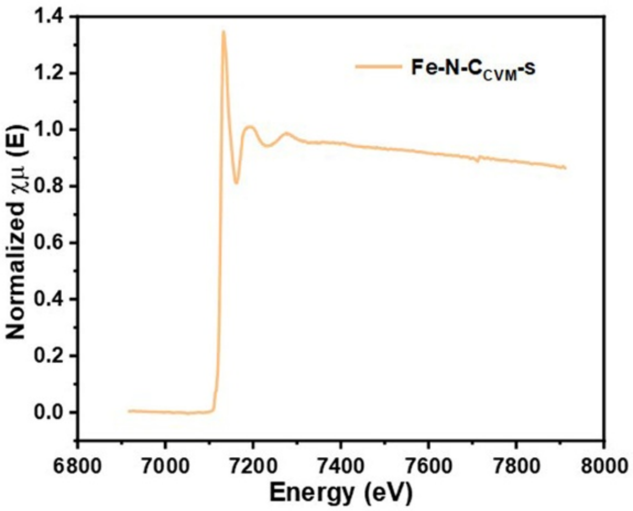

Supplementary Figure 19. | XAS characterization. Normalized XANES spectra of Fe K-edge for Fe-N-C$_{CVM}$-s.

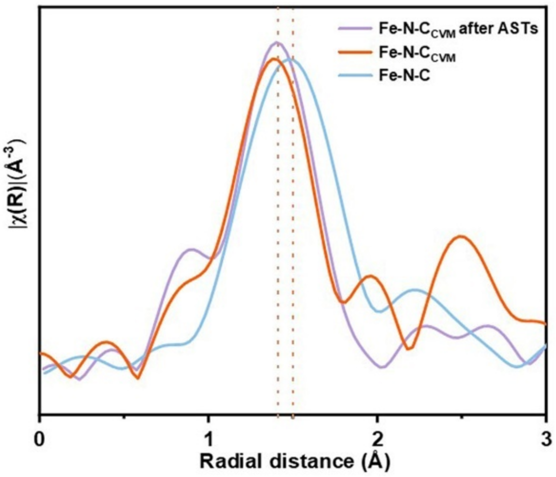

Supplementary Figure 20. | Comparison of R-space for the three electrocatalysts. The k^3-weighted (k)-function of the EXAFS spectra for Fe-N-C_{CVM} after ASTs, Fe-N-C_{CVM}, and Fe-N-C.

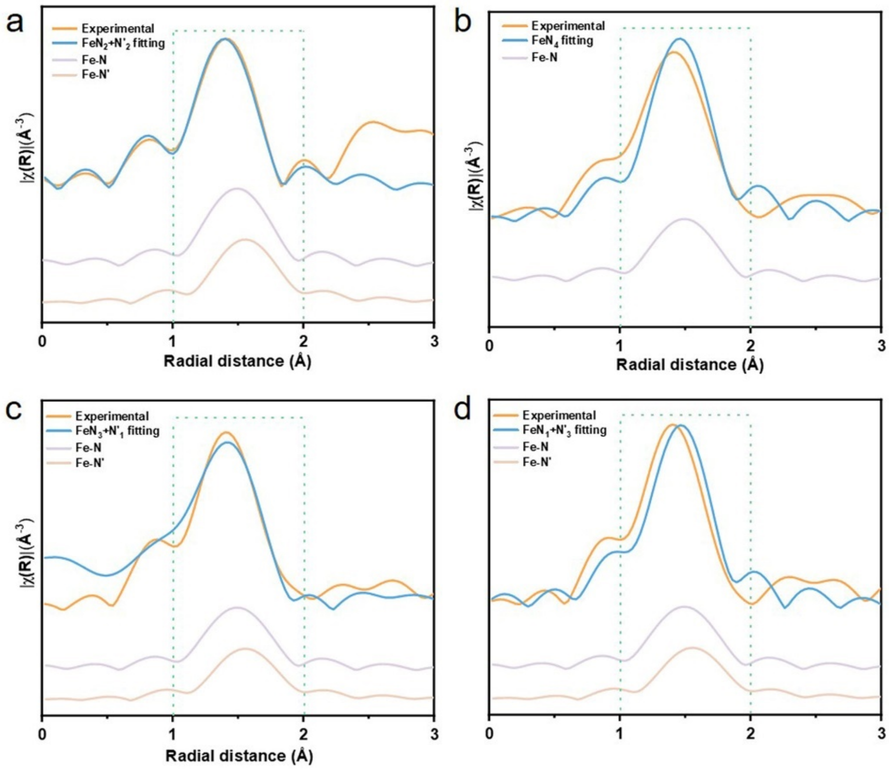

Supplementary Figure 21. | Fine structure analysis. Ex situ Fe K-edge Fourier transform EXAFS spectrum and its fitting with (a) the coordination number evenly distributed in the two paths; (b) four coordination atoms completely filled in a path; (c) first path filled 3 N atoms, the other path filled 1 N atom; (d) first path filled 1, the other path filled 3 N atoms of Fe-N-C_{CVM}.

Supplementary Table 8. Results of the fitting of the FT-EXAFS spectra collected at the Fe-Kedge for Fe-N-C_{CVM}-s (CN: coordination number; R: distance; σ^2: mean-square disorder; ΔE_0: energy shift).

path	CN	R (Å)	ΔE_0 (eV)	$\sigma^2 \times 10^{-3}$ (Å^2)	R factor (%)
Fe-N_2	2	1.95	3.3	4.72	0.009
Fe-N_2’	2	2.05	-0.31	10.00	0.009

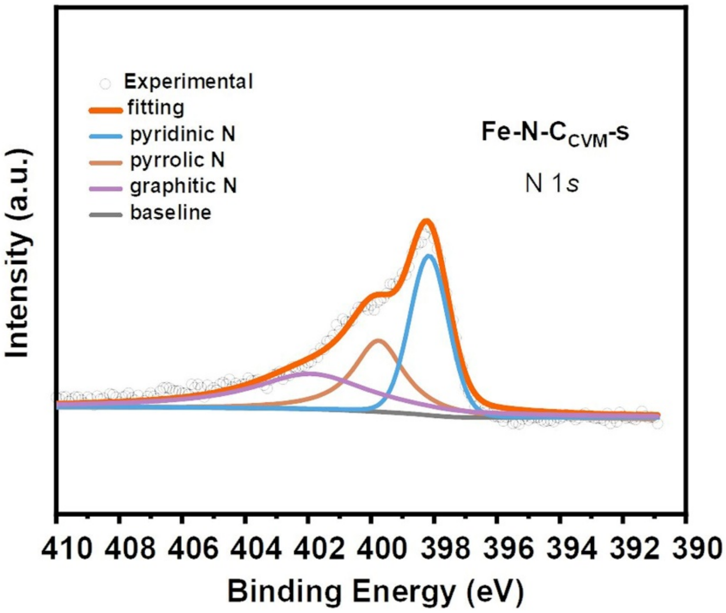

Supplementary Figure 22. | N 1*s* XPS spectra. High-resolution N 1s XPS data of the Fe-N-C_{CVM}-s.

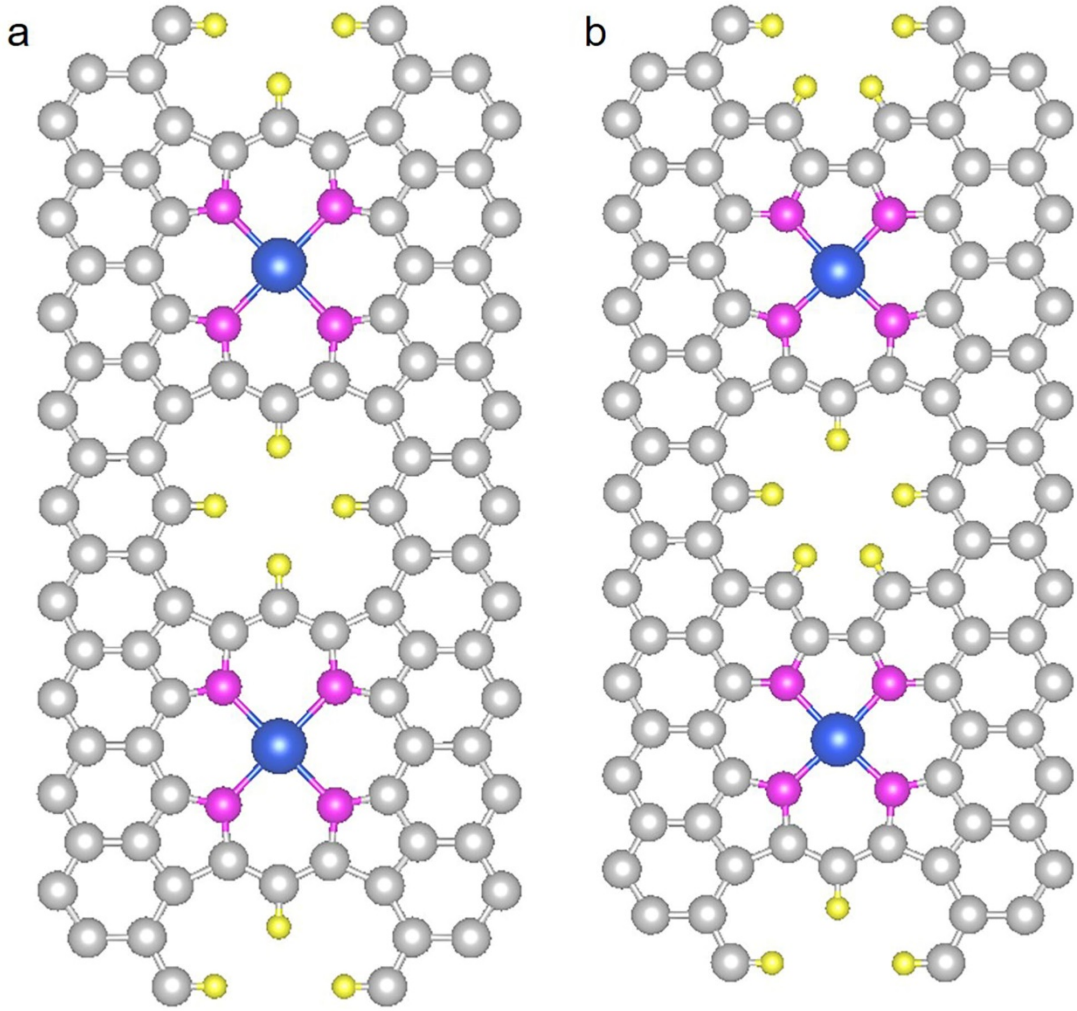

Supplementary Figure 23. | Configuration of active sites. Illustration of (a) FeN_4 site and (b)FeN_2+N'_2 (mix).

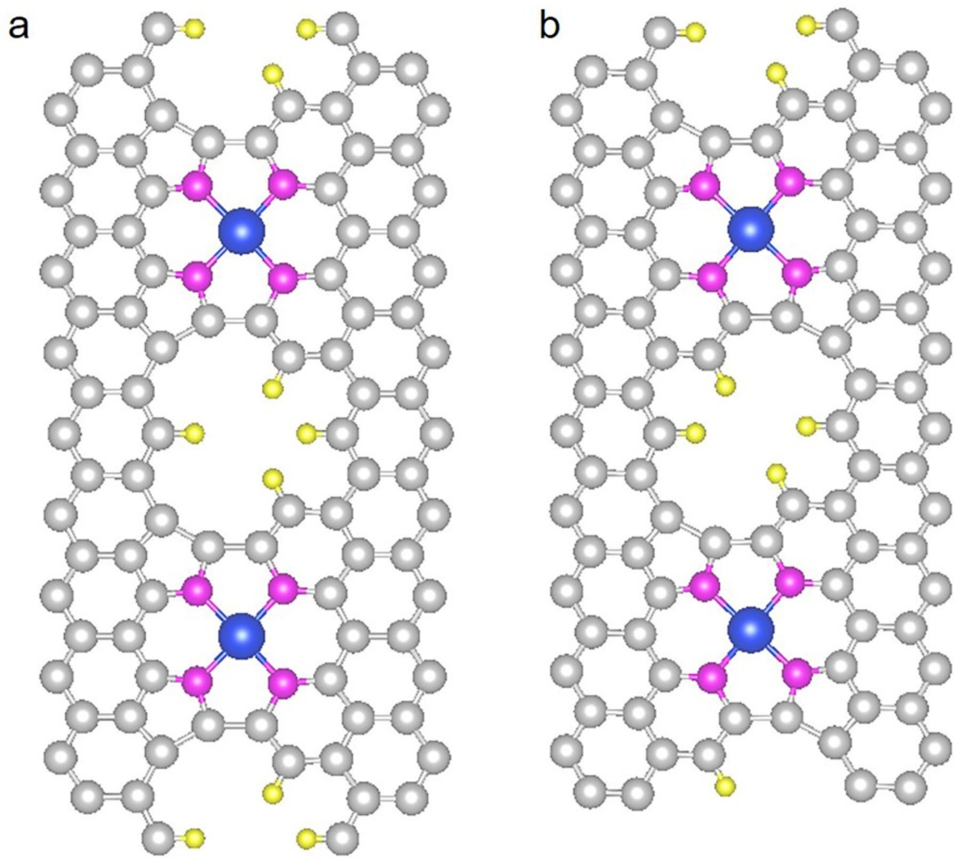

Supplementary Figure 24. | Configuration of active sites. Illustration of (a) mix another and (b) mix third.

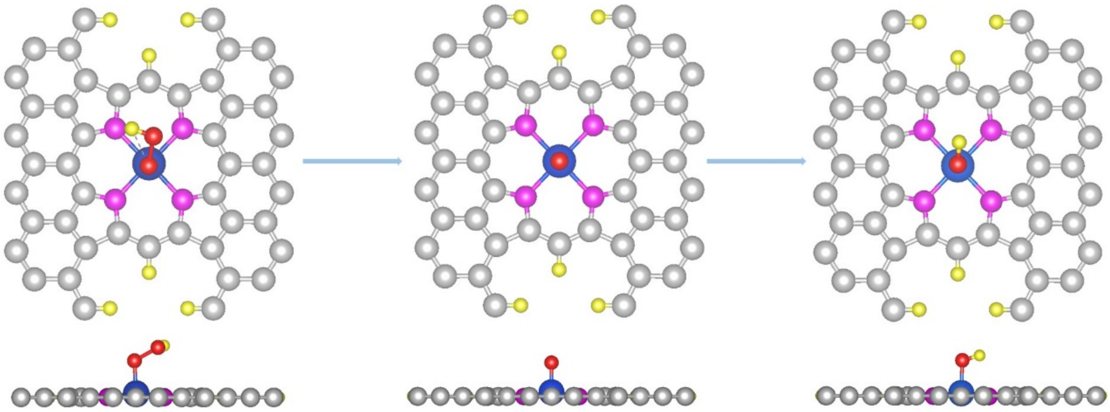

Supplementary Figure 25. | DFT calculations to elucidate the activity of FeN_4 site. ORR mechanism on FeN_4 site**.**

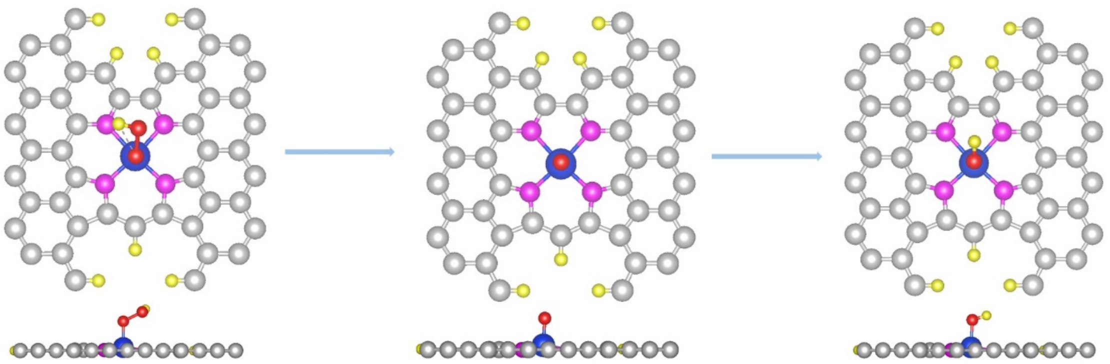

Supplementary Figure 26. | DFT calculations to elucidate the activity of FeN_2+N'$_2$ site. ORR mechanism on FeN_2+N'$_2$ site.

Supplementary Table 9 Formation energy of different active site configurations

configuration	S1	mix	mix another	mix third
E_f	0.728	-1.651	3.179	3.096

References

1. Li J, et al. Atomically dispersed manganese catalysts for oxygen reduction in proton-exchange membrane fuel cells. Nature Catalysis 1, 935-945 (2018).
2. Luo E, et al. Single-Atom Cr-N_4 Sites Designed for Durable Oxygen Reduction Catalysis in Acid Media. Angew. Chem. Int. Ed. Engl. 58, 12469-12475 (2019).
3. Li J, et al. Thermally Driven Structure and Performance Evolution of Atomically Dispersed FeN_4 Sites for Oxygen Reduction. Angew. Chem. Int. Ed. Engl. 58, 18971-18980 (2019).
4. Xie H, et al. Ta-TiO_x nanoparticles as radical scavengers to improve the durability of Fe–N–C oxygen reduction catalysts. Nature Energy 7, 281-289 (2022).
5. Chu Y, et al. Dual single-atom catalyst design to build robust oxygen reduction electrode via free radical scavenging. Chem Catalysis 3, (2023).
6. Fu X, et al. In Situ Polymer Graphenization Ingrained with Nanoporosity in a Nitrogenous Electrocatalyst Boosting the Performance of Polymer-Electrolyte-Membrane Fuel Cells. Adv. Mater. 29, (2017).
7. Wan X, et al. Fe-N-C electrocatalyst with dense active sites and efficient mass transport for high-performance proton exchange membrane fuel cells. Nature Catalysis 2, 259-268 (2019).
8. Li J, et al. Evolution Pathway from Iron Compounds to Fe_1(II)-N_4 Sites through Gas-Phase Iron during Pyrolysis. J. Am. Chem. Soc. 142, 1417-1423 (2020).
9. Xie X, et al. Performance enhancement and degradation mechanism identification of a single-atom Co-N-C catalyst for proton exchange membrane fuel cells. Nature Catalysis 3, 1044-1054 (2020).
10. Jiao L, et al. Chemical vapour deposition of Fe-N-C oxygen reduction catalysts with full utilization of dense Fe-N_4 sites. Nat Mater 20, 1385-1391 (2021).
11. Wang XX, et al. Nitrogen-Coordinated Single Cobalt Atom Catalysts for Oxygen Reduction in Proton Exchange Membrane Fuel Cells. Adv. Mater. 30, (2018).
12. Liu S, et al. Atomically dispersed iron sites with a nitrogen–carbon coating as highly active and durable oxygen reduction catalysts for fuel cells. Nature Energy 7, 652-663 (2022).
13. Yang Z, et al. Boosting Oxygen Reduction Catalysis with Fe-N_4 Sites Decorated Porous Carbons toward Fuel Cells. ACS Catalysis 9, 2158-2163 (2019).
14. Liu S, et al. Chemical Vapor Deposition for Atomically Dispersed and Nitrogen Coordinated Single Metal Site Catalysts. Angew. Chem. Int. Ed. Engl. 59, 21698-21705 (2020).
15. Shao Y, et al. PGM-Free Cathode Catalysts for PEM Fuel Cells: A Mini-Review on Stability Challenges. Adv. Mater. 31, e1807615 (2019).
16. Wan X, et al. Iron atom-cluster interactions increase activity and improve durability in Fe-N-C fuel cells. Nat Commun 13, 2963 (2022).
17. Yin S-H, et al. Seizing gaseous Fe^{2+} to densify O_2-accessible Fe-N_4 sites for high-performance proton exchange membrane fuel cells. Energy & Environmental Science 15, 3033-3040 (2022).
18. Cheng X, et al. Instantaneous Free Radical Scavenging by CeO_2 Nanoparticles Adjacent to the Fe-N_4 Active Sites for Durable Fuel Cells. Angew. Chem. Int. Ed. Engl.

62, e202306166 (2023).
19. Im K, et al. Design of Co-NC as efficient electrocatalyst: The unique structure and active site for remarkable durability of proton exchange membrane fuel cells. Applied Catalysis B: Environmental 308, (2022).
